# Elucidating the diversity of malignant mesenchymal states in glioblastoma by integrative analysis

**DOI:** 10.1186/s13073-022-01109-8

**Published:** 2022-09-19

**Authors:** Rony Chanoch-Myers, Adi Wider, Mario L. Suva, Itay Tirosh

**Affiliations:** 1 Department of Molecular Cell Biology, Weizmann Institute of Science, Rehovot, Israel; 2 Department of Pathology and Center for Cancer Research, Massachusetts General Hospital, Boston, MA USA; 3 Broad Institute of MIT and Harvard, Cambridge, MA USA

## Abstract

**Background:**

Multiple glioblastoma studies have described a mesenchymal (MES) state, with each study defining the MES program by distinct sets of genes and highlighting distinct functional associations, including both immune activation and suppression. These variable descriptions complicate our understanding of the MES state and its implications. Here, we hypothesize that there is a range of glioma MES states, possibly reflecting distinct prior states in which a MES program can be induced, and/or distinct mechanisms that induce the MES states in those cells.

**Methods:**

We integrated multiple published single-cell and bulk RNA sequencing datasets and MES signatures to define a core MES program that recurs across studies, as well as multiple function-specific MES signatures that vary across MES cells. We then examined the co-occurrence of these signatures and their associations with genetic and microenvironmental features.

**Results:**

Based on co-occurrence of MES signatures, we found three main variants of MES states: hypoxia-related (MES-Hyp), astrocyte-related (MES-Ast), and an intermediate state. Notably, the MES states are differentially associated with genetic and microenvironmental features. MES-Hyp is preferentially associated with NF1 deletion, overall macrophage abundance, a high macrophage/microglia ratio, and M2-related macrophages, consistent with previous studies that associated MES with immune suppression. In contrast, MES-Ast is associated with T cell abundance and cytotoxicity, consistent with immune activation through expression of MHC-I/II.

**Conclusions:**

Diverse MES states occur in glioblastoma. These states share a subset of core genes but differ primarily in their association with hypoxia vs. astrocytic expression programs, and with immune suppression vs. activation, respectively.

**Supplementary Information:**

The online version contains supplementary material available at 10.1186/s13073-022-01109-8.

## Background

IDH-WT glioblastoma (GBM) is the most aggressive primary brain tumor, with no effective cure. GBM is known to exhibit a high degree of inter- and intra-tumor heterogeneity [1–4] and many recent studies have used bulk or single-cell RNA-seq (scRNA-seq) to characterize this heterogeneity. A central and consistent observation across these studies is the existence of mesenchymal signatures. The Cancer Genome Atlas (TCGA) analysis first classified GBM into 3 subtypes (classical, proneural, and mesenchymal) [1, 2, 5]. More recently, multiple studies applied single-cell RNA-seq to GBM patient samples, or model systems, and consistently identified a MES state of malignant cells [3, 4, 6–10]. The GBM MES states involve genes which are normally expressed by non-cancerous mesenchymal cells such as fibroblasts, and by cells undergoing epithelial-to-mesenchymal transition (EMT) during neural tube formation or wound healing [11]. EMT and similar processes [12] are co-opted by many cancer types, in which they are associated with metastasis, tumor aggressiveness, and resistance to therapies [13, 14].

Despite the consistent observation of MES states by many GBM studies, our understanding of these states remains very partial. The origin, regulation, functions, interactions with other cells, and clinical implications of MES states are yet unclear. A central difficulty in addressing these questions involves the distinct characterization of the MES states by different studies. First, the set of signature genes that define the MES state differs considerably between studies and it is difficult to conclude on any specific set of genes as representing the MES state. Second, multiple factors have been proposed as master regulators of the MES states, including TAZ, STAT3, CEBP, OSMR, NFkB, and HIF-1a [15]. The connections between these regulators and thei relative significance of these regulators in driving the MES states are unclear. Third, specific studies have emphasized the association of MES states with hypoxia [4, 16], with treatment response [17, 18], with glioma stem cells [19], and with either immune suppression [20] or with immune activation [6]. The MES states of GBM have consistently been associated with the abundance of macrophage/microglia cells [2] but the association of MES states with T cells differs between studies. We have recently shown that the MES state is associated with high expression of MHC-I/II genes and with increased cytotoxicity of T cells [6]. However, another recent study of GBM mice models demonstrated that MES states were associated with the expression of interferon-response genes and consequently with suppression of T cells [20]. This discrepancy highlights the complex relationship between MES states and T cells and the possibility that there are various flavors of MES states with distinct interactions and implications.

Taken together, the discrepancy between studies, the potential involvement of MES states in a wide range of functions, its prognostic significance, and the potential for multiple flavors of MES states call for an integrative analysis of MES states. To address these issues, in this work we contrasted 10 GBM MES signatures from previous studies (Additional file 1: Table S1) [1, 2, 4, 6, 8–10, 20]. We explored the co-expression of these signatures in a cohort of 56 patients combined from 4 publicly available GBM scRNAseq datasets to define MES states: [24 tumors published in [4], 14 tumors published in [10], 10 tumors published in [8], and 8 tumors published in [9] (Additional file 1: Table S2). Finally, we expanded our analysis of the MES states to bulk RNA-seq by leveraging the TCGA and Ivy-GAP datasets [2, 21–23] to examine associations with microenvironmental and genetic factors. We identify three main states of mesenchymal GBM cells with distinct genetic and environmental associations: (1) a MES state that expresses a hypoxia program and is associated with NF1 deletion, macrophage abundance, an increased macrophage to microglia ratio, and M2 activated macrophages; (2) a MES state that expresses astrocytic genes and is associated with T cell abundance and cytotoxicity; and (3) an intermediate state which may represent their parental state.

## Methods

### Cohorts used in this study

We collected GBM MES signatures from the following studies: [1, 2, 4, 6, 8–10, 20] (Additional file 1: Table S1).

We performed the analysis examining the functional MES signatures to define the MES states in data from a total of 56 IDH-WT GBM patients that met our requirements of at least 50 malignant cells that had at least 2000 detected genes. The data from these samples were obtained from 4 publicly available GBM scRNAseq datasets: [24 tumors from [4], 14 tumors from [10], 10 tumors from [8], and 8 tumors from [9]]. The MES core distribution was also explored in 2 IDH-mutant datasets [24, 25] and 1 H3K27M glioma dataset [26]. Details on all samples and their original published cohorts are described in Additional file 1: Table S2.

We performed the analysis examining the associations of MES states with genetic and microenvironmental associations using publicly available bulk RNAseq data from the TCGA. We performed the analysis examining the spatial distributions of the MES states using the Ivy-GAP dataset [21].

### Generating MES-core and MES functional programs

The MES-core signature was defined as all genes that (i) were overlapping in at least 3 of the 10 signatures we collected, and (ii) had an annotation related to mesenchymal cells. The remaining genes were assigned into functional programs using annotations of enriched Gene Ontology (GO) terms. Genes that had no annotation for any of the enriched signatures were excluded from further analysis.

### ScRNA-seq data processing

Single-cell RNA-seq was downloaded from published datasets in *TPM* or *UMI* count matrices. First, using available metadata from each study, non-malignant cells and cells from samples with fewer than 50 malignant cells were excluded. Next, as quality control, we excluded cells with fewer than 2000 detected genes. For *TPM* matrices, expression levels were quantified as *E(I,j) = log2(TPM(i,j)/10+1)*, where *TPM(i,j)* refers to transcript-per-million for gene *i* in cell *j*, as quantified by RSEM [27]. The average number of transcripts detected per cell was less than 100,000, thus *TPM* values were divided by 10, to avoid inflating the differences between detected (*E(i,j) > 0*) and non-detected (*E(i,j) = 0*) genes, as previously described [24]. For UMI count matrices, expression levels were quantified as *E(i,j) = log2(1 + CPM(i,j)/10)*, where *CPM(i,j)* refers to *10*^*6∗*^*UMI(i,j)/sum[UMI 1..n,j]*, for gene *i* in sample *j*, with n being the total number of analyzed genes. *CPM* values were divided by 10, as described above for *TPM* values. We defined relative expression over the remaining cells for each study separately, by centering the expression levels per gene, *E*_*rel*_*(i,j) = Ei,j–- mean[Ei,1...n]*.

### Definition of gene signature scores

Cells or bulk tumors were scored for a gene signature as previously described [4], using the R package scalop [28]. Given a set of genes (*Gj*) reflecting an expression signature of a specific cell type or biological function, we calculate for each cell *i*, a score, *SCj (i)*, quantifying the relative expression of *Gj* in cell *i*, as the average relative expression (*Er*) of the genes in *Gj*, compared to the average relative expression of a control gene-set (*Gj* cont): *SCj (i) = average[Er(Gj,i)] – average[Er(Gj cont,i)]*. The control gene-set is defined by first binning all analyzed genes into 30 bins of aggregate expression levels (*Ea*) and then, for each gene in the gene-set *Gj*, randomly selecting 100 genes from the same expression bin. In this way, the control gene set has a comparable distribution of expression levels to that of *Gj*, and the control gene set is 100-fold larger, such that its average expression is analogous to averaging over 100 randomly selected gene sets of the same size as the considered gene-set. Cells were scored for each study separately.

### Assignment of cells as MES

For each study, the *E*_*avg*_ matrix was used to score the cells for the MES-core program as described above. In addition, the *E*_*avg*_ matrix values were shuffled and used to score the cells for the core MES program. All cells that had a core MES score above the 99% quantile of the shuffled MES score were assigned as MES. In addition, to compare proportions of MES cells between glioma types, we used a global threshold of *1* for the core MES program. For inter-patient analyses, only patients with at least 20 MES cells were included.

### Principal component analysis of MES cells

We performed PCA for the expression values of the MES cells per study. To decrease the impact of inter-tumor variability on the combined analysis of the cells, we recentered the data within each tumor separately. Finally, we compared the genes that had the highest correlations to each of the first 3 PCs across studies to identify the consistent effects.

### Bulk scores defined for TCGA and IVY-GAP samples

Expression data from bulk samples using the RNA-sequencing platform were log-transformed and centered by the expression levels per gene.

### Association of MES states with macrophage and T cell states in TCGA

To evaluate if MES states are associated with a specific subtype of macrophages or T cells, we used the bulk expression profiles from TCGA. First, we used our glioblastoma scRNA-seq data to compare the average expression of each gene across different cell types and identified genes that were at least 8-fold higher expressed in macrophages or T cells than in any other cell type, as previously described [6]. Next, we scored each tumor in the TCGA dataset for the glioblastoma core MES program, and for their total macrophage or T cell signal, defined by a set of canonical marker genes for macrophages (CD14, AIF1, CD163, TYROBP, CSF1R) or for T cells (CD2, CD3D, CD3E, and CD3G). For the scatter plots in Fig. 3A and B, we plotted each gene expression’s correlation to the core MES score against its correlation to the total macrophage or T cell score.

### Partial correlation of MES-Hyp and MES-Ast with NF1 expression, macrophage, and T cell states

To evaluate which MES state was associated with NF1 expression or the abundance\specific subtype of macrophages or T cells, we used the bulk expression profiles from TCGA. For each of the factors examined, the partial correlation of their score with the core MES score was calculated and compared to their correlation to the core MES score. The robustness of this comparison was tested by repeating this correlation 1000 times where 75 out of 150 tumors were sampled each time and comparing the medians using Wilcoxon ranked sum test.

## Results

### Integrative analysis defines core and function-specific MES programs

**Fig. 1:**
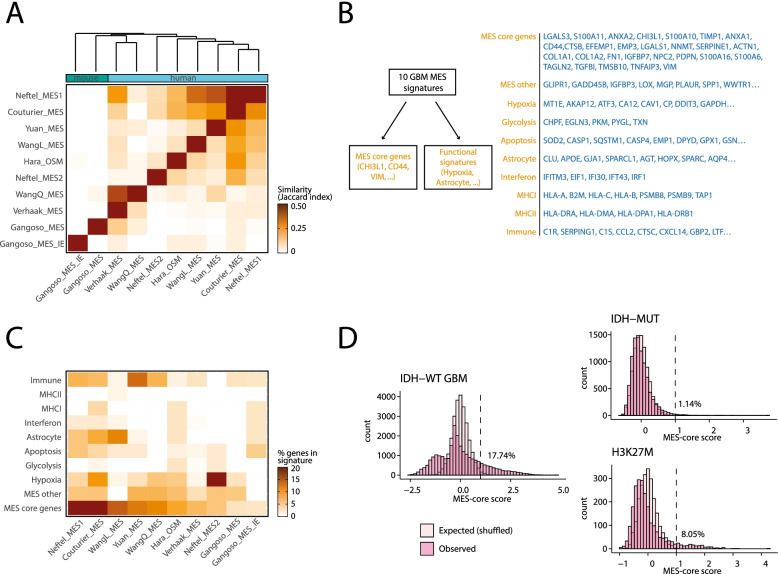
Defining the core and function-specific MES programs. **A** Heatmap shows 10 GBM MES signatures clustered by their similarity as defined by Jaccard Index. Annotation bar shows the human or mouse origin of each signature. Signature names include the first author of the associated study; Gangoso_MES_IE represents the immune evasion signature from Gangoso et al.; Hara_OSM represents the signature of upregulated genes after GBM cells were treated with OSM in Hara et al. **B ***Left*: scheme explaining how the MES functional programs and core MES signatures were defined. *Right*: gene lists of all novel MES signatures (see full list in Additional file 1: Table S3). **C** Heatmap depicting the percentage of genes from each core or function-specific MES signature (*Y*-axis) that are included in each of the published MES signatures (*X*-axis). Published signatures are ordered by the percentage of genes from the core MES program. **D** Distributions of the core MES scores (pink) of cells from IDH-WT GBM (*left*), IDH-mutant glioma (*top right*), and H3-K27M glioma (*bottom right*). The light color represents core MES scores of the shuffled dataset. A threshold of 1 and the percentage of cells that pass it are also included in each panel

We collected 10 GBM MES signatures from distinct studies and clustered them based on the degree of overlap among signature genes (Fig. 1A, Additional file 1: Table S1) [1, 2, 4, 6, 8–10, 20]. Overall, there was limited overlap between studies. Many pairs of signatures had zero overlap, and the average overlap across all pairs was only 0.091 by the Jaccard index (the number of genes shared by both signatures divided by the total number of genes across the two signatures combined). Despite this low overlap, 45 genes were consistently observed across at least 3 studies, and these include canonical markers of mesenchymal cells, such as Vimentin (VIM), Fibronectin (FN1), Podoplanin (PDPN), and collagen (COL1A1/2) as well as genes frequently noted as key markers of glioma MES (CHI3L1 and CD44). We therefore defined the 28 genes that (i) appear in at least three distinct MES signatures and (ii) have a mesenchymal-related functional annotation, as the *core* MES program, which we suggest should be used by future studies as a more robust definition for MES than any of the individual signatures published previously.

The remaining genes either appear in only one/two signatures or are not known to associate with mesenchymal cells. To better characterize the functions associated with those genes, we identified frequent functional annotations and defined non-overlapping sets of genes with each of those annotations. These function-specific signatures represent different aspects of MES states, including hypoxia, glycolysis, astrocytic lineage, interferon response, MHC-I and MHC-II (Fig. 1B, Additional file 1: Table S3). Two additional function-specific signatures include those with an immune-related annotation that does not fit into the specific immune functions noted above (interferon or MHCI/II) and those with a mesenchymal-related annotation that is included only in one or two signatures and hence are not part of the core MES signature. Each of the 10 MES signatures described by previous studies contained a combination of genes from the core MES and from multiple function-specific MES programs, with varying biases towards particular programs (Fig. 1C). For example, Neftel MES2 had more genes from the hypoxia signature compared to other signatures, while the WangL-MES had more genes from the Astrocytic signature.

### Identifying MES cells across glioma cohorts

Next, we utilized the core MES signature to comprehensively identify MES cells in a pan-study cohort of glioma scRNA-seq. We combined 4 published scRNAseq datasets of IDH-WT GBM [4, 8–10] and removed tumors with less than 50 malignant cells that pass our QC. The resulting dataset included 56 IDH-wild type GBMs (52 adult and 4 pediatric). In addition, we included 2 studies of IDH-mutant gliomas [24, 25] containing 6 oligodendrogliomas and 10 astrocytomas, and 1 study of 6 pediatric brainstem H3-K27M gliomas [26]. Additional details on each patient and study can be found in Additional file 1: Table S2.

To identify MES cells in each of these gliomas, we scored all cells for expression of the core MES program and compared the observed MES scores in each study to the scores derived from a control dataset generated by shuffling that dataset (Fig. 1D, Additional file 2: Figs. S1A-B). Malignant cells were defined as MES if their scores were higher than the 99% quantile of the control dataset and also higher than a score of 1 (i.e. 2-fold higher than the average expected score). In the GBM cohort, 17.74% of cells were identified as MES. In contrast, samples from IDH-mutant glioma had a much lower proportion of MES cells (1.14%). Moreover, those few IDH-mutant MES cells only expressed a small subset of the core MES program, unlike the GBM MES cells that expressed most of the core MES program (Additional file 2: Fig. S1C). An intermediate fraction of MES cells was identified in H3-K27M gliomas (8.05%), with robust expression of the core MES program that was more reminiscent of the GBM than the IDH-mutant MES cells. We conclude that MES cells are abundant in GBM, occur at a lower frequency in H3-K27M glioma, and are practically absent in IDH-mutant glioma. However, since our analysis is driven by the MES signatures of IDH-WT GBM, we cannot exclude the possibility of a distinct MES program in IDH-mutant glioma.

The mesenchymal subtype was previously shown to be a poor prognostic indicator in a cohort of high-grade astrocytomas which included both IDH WT and mutant gliomas [29]. However, through reanalysis of the TCGA high-grade glioma dataset, we noticed that the difference in survival between the MES and non-MES subtypes is mainly due to the difference between IDH-mutant and IDH-WT tumors (as MES cells are depleted in IDH-mutant glioma) and is no longer significant when the analysis is restricted to IDH-WT GBMs (Additional file 2: Fig. S1D).

### Co-occurrence of programs defines three flavors of MES states

**Fig. 2:**
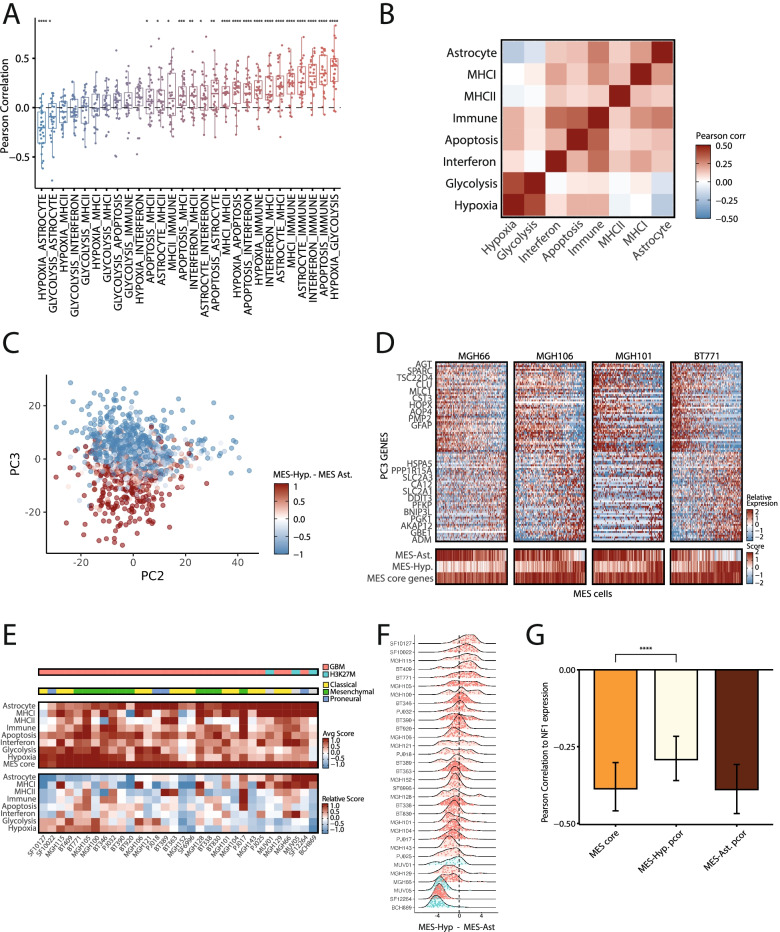
Three MES states of GBM. **A** Boxplots show the Pearson correlations of the scores of each pair of GBM function-specific signatures across MES cells, where each dot represents the correlation in one patient. Significance defined by t-test is indicated with asterisks (∗∗∗∗, *p* < 0.0001; ∗∗∗, *p* < 0.001; ∗∗, *p* < 0.01; *, *p* < 0.05). In each boxplot, horizontal line shows the median, the box borders show upper and lower quartiles, the vertical line indicates the 5th through 95th percentiles. **B** Heatmap depicts the Pearson correlation between pairs of MES programs, averaged across patients. **C** Dot plot shows the PC2 and PC3 scores of MES cells from all of the samples from the Neftel et al. dataset, colored by the difference between their score for MES-Hyp and MES-Ast. **D ***Top*: Heatmaps of 4 samples from Neftel et al. *X*-axis represents MES cells ordered by their PC3 score and *Y*-axis shows highest and lowest scoring PC3 genes. Selected genes from MES-Ast (top) or MES-Hyp (bottom) are labeled. *Bottom*: Heatmap shows the average score of each cell for the core MES, MES-Hyp, and MES-Ast programs. **E** Main heatmaps show the score of the MES cells from each tumor for the different MES programs. The top heatmap shows the average (uncentered) score and the bottom heatmap shows the score after centering each row (across samples). Annotation bars show the glioma type (top) or the sample’s TCGA-subtype (bottom). **F** Distributions of the difference in MES-Hyp and MES-Ast scores for individual tumors, colored by glioma type as in **E**). **G** Bars show the correlation between the expression of NF1 and the core MES scores (orange), and the partial correlation of NF1 expression with core MES scores after controlling for MES-Hyp scores (yellow) or for MES-Ast scores (brown), across TCGA bulk RNA-seq samples. Error bars correspond to first and third quartiles based on sampling 75 tumors with 1000 iterations; the difference between averages is significant by Wilcoxon rank sum test (*p*=2.2e−16) as indicated by asterisks

The function-specific programs constitute a variety of features that characterize only subsets of MES cells. One possibility is that these features are independent of one another. Alternatively, these features might be inter-dependent such that certain combinations are more or less common than expected by chance, thus creating consistent flavors of MES cells. To examine this possibility, we explored the correlations between the expression of each pair of function-specific programs in MES cells from the GBM cohort (Fig. 2A, B).

Most pairs of function-specific programs (20 of 28) were mildly, yet significantly, positively correlated, suggesting an overall tendency of different MES features to be co-expressed. However, two pairs were significantly negatively correlated – Astrocyte with Hypoxia and Astrocyte with Glycolysis. Notably, Hypoxia and Glycolysis were the most highly positively correlated pair, consistent with a metabolic shift of GBM cells from oxidative phosphorylation to glycolysis in the absence of oxygen. Taken together, these results suggest two distinct MES flavors, one associated with Astrocytes (MES-Ast) and the other with Hypoxia+Glycolysis (MES-Hyp) (Additional file 2: Fig. S2A). The remaining functional programs were either equally correlated with both MES-Hyp and MES-Ast (Immune, Interferon, and Apoptosis) or were preferentially associated with MES-Ast (MHC-I/II). This latter association may suggest that increased recognition and killing of MES cells by T cells [6] may be restricted to the MES-Ast.

To further evaluate the separation of GBM MES cells into distinct flavors, we utilized a distinct computational approach—an unbiased principal component analysis (PCA) of all the GBM MES cells. PC1 primarily separated the cells by their number of detected genes (Additional file 2: Fig. S2B), suggesting a technical effect. However, PC2 and primarily PC3 effectively distinguished between MES-Ast and MES-Hyp across multiple studies (Fig. 2C, D, Additional file 2: S2C, Additional file 1: Table S4), supporting the robustness of our results and of the two MES flavors.

Next, we wondered if individual GBM tumors tend to have only one of the MES flavors or whether the two flavors tend to co-exist within the same tumor. To address this, we defined a differential score for the two flavors (i.e., MES-Hyp score minus and MES-Ast score) and examined the distribution of these differential scores across MES cells from each patient (Fig. 2E, F, Additional file 2: S2D). Approximately half of the tumors had a wide bi-modal (or multi-modal) distribution suggestive of considerable co-existence of MES states. The other half of the samples had a more narrow distribution with one apparent peak, although each of these tumors still harbored a considerable range of differential scores. H3K27M tumors were highly skewed to the MES-Ast program, suggesting that MES-Hyp is largely specific to GBM. Among GBMs, the bias of tumors towards specific MES states was not correlated with different GBM subtypes and most GBMs peaked at intermediate differential scores, indicating that most MES cells do not display a considerable bias towards either the hypoxia or astrocytic state. We therefore defined a third MES flavor, representing an intermediate state between MES-Hyp and MES-Ast that includes the majority of MES cells (Additional file 2: Fig. S2A).

To understand the spatial distributions of the MES flavors in a given tumor, we leveraged the Ivy Glioblastoma Atlas Project (Ivy-GAP) [21] (Additional file 2: Fig S2E). Samples that expressed both core MES and MES-Hyp were almost solely in the “Pseudopalisading cells around necrosis” region, as expected since this region is known to be hypoxic. Samples that expressed both core MES and MES-Ast were mainly in the “Cellular Tumor” region and few samples from the “Infiltrating Tumor”. In addition, the “Microvascular proliferation” region expressed core MES genes but not either of the MES-Hyp or MES-Ast  programs. Taken together, the three MES flavors tend to co-exist within tumors, although unique tumor regions tend to be enriched with particular states.

Next, we explored if the tumor-specificity of MES states is linked to genetics. The mesenchymal subtype is associated with NF1 deletion [2]. Accordingly, NF1 expression is negatively correlated with bulk tumor MES scores across the TCGA GBM cohort (Fig. 2G, Additional file 2: S2F). To examine if NF1 expression is associated with a particular flavor of MES states, we tested how the correlation is affected by controlling for the signatures of MES-Hyp and MES-Ast through partial correlations (Fig. 2G). We found that the partial correlation of the core MES score and NF1 expression decreased when controlling for the MES-Hyp score but not when controlling for the MES-Ast score, suggesting that NF1 deletion is preferentially associated with the MES-Hyp state.

### Association of MES states with macrophage abundance and states

**Fig. 3:**
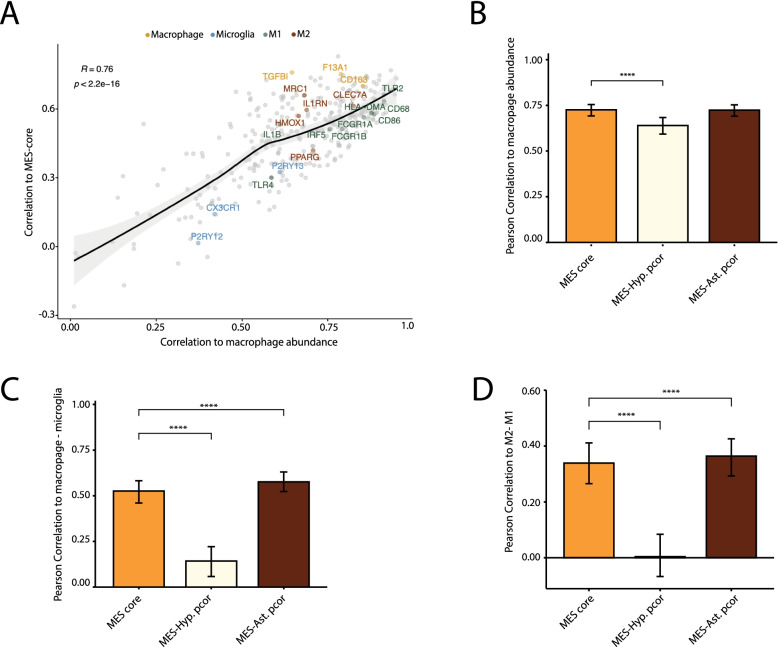
MES association with macrophage states. **A** Scatterplot showing the correlations of 340 macrophage-specific genes (dots) with estimated macrophage abundance (*X*-axis), as defined by average expression of canonical macrophage markers (CD14, AIF1, CD163, TYROBP, CSF1R) and with core MES scores (*Y*-axis), across TCGA bulk RNA-seq samples. Line indicates a LOESS regression and a confidence interval is shown in grey. Dot colors indicate marker genes for macrophage subtypes (microglia, macrophage, M1 activation, M2 activation). **B**–**D** Bars show correlations or partial correlations of the core MES scores with macrophage-related scores: macrophage abundance as defined above (**B**), the difference between macrophage and microglia scores (**C**) or the difference between M2 and M1 scores (**D**). Partial correlations are included to control for MES-Hyp (yellow) or MES-Ast (brown) scores. Error bars correspond to Q1 and Q3, calculated by bootstrapping 75 tumors with 1000 iterations; the difference between averages is significant by Wilcoxon rank sum test (**** *p*< 2.2e−16)

The MES state is associated with infiltration of macrophages, and we have recently shown that macrophages can directly induce the MES state by secretion of OSM [6]. Accordingly, we found a positive correlation between the core MES score and macrophage abundance in TCGA data (Additional file 2: Fig. S3A). To understand the specific macrophage state associated with core MES program, we examined the correlations of all macrophage-specific genes with the core MES program (Fig. 3A). Genes with higher correlations to macrophage abundance also had higher correlations to the MES-core scores of bulk tumors, again reflecting the overall association between macrophage abundance and MES. We estimated this expected trend with a LOESS regression and examined which of the macrophage genes have higher or lower correlations with the core MES score compared with the regression. We found that macrophage markers (e.g., CD163, F13A1, TGFBI) and M2-related activation markers (e.g. CLEC7A, MRC1) had higher correlations, while microglia markers (e.g. CX3CR1, P2RY13, P2RY12) and M1-related activation markers (e.g. CD68, CD86, HLA-DMA) had lower correlations with the core MES scores. To better understand which flavor of MES state is associated with macrophage abundance and states, we tested how the correlation of macrophages with core MES score was affected by controlling for specific MES states. The correlation of core MES with overall macrophage abundance decreased when controlling for MES-Hyp score but not when controlling for MES-Ast score (Fig. 3B). In addition, the partial correlation of core MES with the macrophages-to-microglia ratio and with M2 vs. M1 scores decreased when controlling for the MES-Hyp score and increased when controlling for the MES-Ast score (Fig. 3C, D, Additional file 2: Figs. S3B-C). Taken together, these results suggest that the MES-Hyp state is preferentially associated with a higher ratio of macrophages to microglia and the M2 state.

### Mesenchymal association with T cell abundance and cytotoxicity

**Fig. 4:**
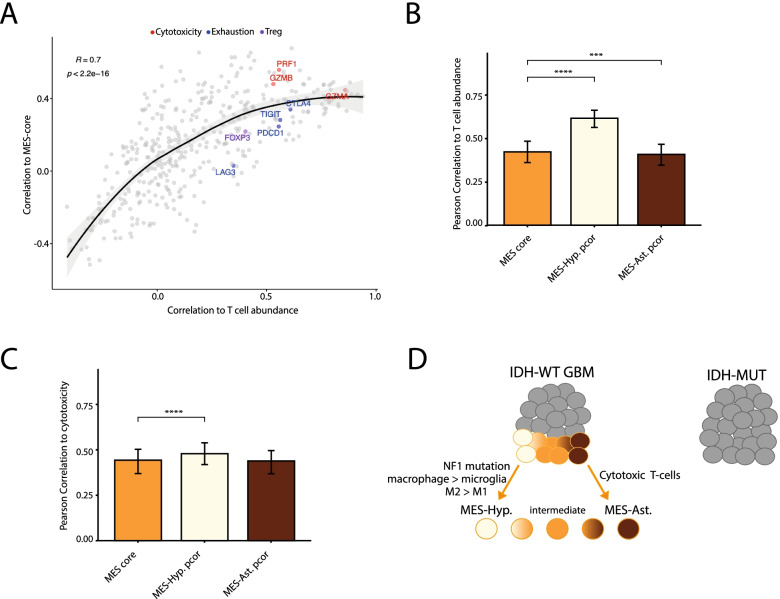
MES-like states association with T cell states. **A** Scatterplot showing the correlations of 406 T cell-specific genes (dots) with estimated T cell abundance (*X*-axis), as defined by average expression of canonical marker genes (CD2, CD3D, CD3E, CD3G), and with the core MES score (*Y*-axis), across TCGA bulk RNA-seq samples. Line indicates a LOESS regression and confidence intervals are shown in grey. Dot colors indicate marker genes for T cell subtypes (cytotoxic, Treg, exhaustion). **(B-C)** Bars show correlations or partial correlations between core MES scores and T cell-related scores: T cell abundance as defined above (**B**), or the difference between cytotoxicity and exhaustion scores (**C**). Partial correlations control for the MES-Hyp (yellow) or MES-Ast (brown) scores, across TCGA bulk RNA-seq samples. Error bars correspond to first and third quartiles, calculated by sampling 75 tumors with 1000 iterations; the difference between averages is significant by Wilcoxon rank sum test (***, *p* = 0.0003; **** *p*< 2.2e−16). **D** Model depicting the three MES states in GBM. Cells in IDH-WT GBM (left) can be found in four states, one of them is the MES state (colored), while IDH-mutant gliomas (right) do not contain MES cells. MES cells all express a core MES program and can be found in three main flavors: a MES-Hyp state (light yellow), MES-Ast state (brown), and intermediate states (orange). The MES-Hyp state is associated with macrophage abundance and a high macrophage-microglia and M2-M1 activation. The MES-Ast state is associated with higher T cell abundance and T cell cytotoxicity

In addition to macrophages, an association between MES cells and T cells has been previously described [6]. Like macrophages, we found a positive correlation between the core MES program and T cell abundance in TCGA (Additional file 2: Fig. S4A). To understand which T cells wa correlate with MES, we investigated the correlations of T cell-specific genes with the MES-core program. We found that cytotoxicity genes (*PRF1, GZMB*) had higher correlations to the MES-core program while exhaustion (*LAG3*, *PDCD1*, *TIGIT*) and Treg (*FOXP3*) markers had lower correlations with the core MES program (Fig. 4A), as previously described [6]. A potential caveat of this analysis is that expression of cytotoxicity and/or exhaustion markers may not accurately reflect T cell phenotypes and, for example, could reflect negative feedback following T cell activation. To understand which MES state was associated with T cell abundance and cytotoxicity, we tested how the correlation was affected by controlling for MES-states. We found that the partial correlation of core MES scores and T cell abundance increased when controlling for MES-Hyp scores but not when controlling for MES-Ast score (Fig. 4B). The partial correlation between MES-core and T cell cytotoxicity increased, albeit only slightly, when we controlled for the MES-Hyp score (Fig. 4C, Additional file 2: S4B), suggesting that MES-Ast is preferentially associated with T cell activation. Accordingly, MHC I and MHC II were correlated with the MES-Ast program in the previous analysis of scRNAseq data (Fig. 2A).

## Discussion

Through integrative analysis of GBM datasets and signatures, we showed that there is a range of MES states in GBM and highlighted three MES flavors with distinct functional, genetic, and environmental associations (Fig. 4D). One MES flavor is associated with hypoxia, a central process in GBM [15, 30], likely reflecting a hypoxia-driven transition of cells into the MES states [31–33]. A second MES flavor is associated with the astrocytic program, perhaps reflecting the origin of many MES cells from astrocytic-like malignant cells. The third flavor reflects an intermediate between the above two flavors, highlighting the continuous nature of cell states.

Our model may explain the discrepancy between our previous work [6] and Gangoso et al [20] regarding the association of MES cells with T cells. The MES-Ast state is associated with MHC-I and MHC-II expression as well as an abundance of T cells, skewed to cytotoxicity, suggesting that this state promotes T cell activation. On the other hand, MES-Hyp is associated with an abundance of macrophages, skewed towards immunosuppressive M2 activation [34], therefore suggesting that MES-Hyp can lead to suppression of T cells. The co-existence of MES states in tumors, the inter-patient heterogeneity of their proportions, as well as the existence of an intermediate MES state suggest that the T cell response relating to MES is complex and would need to be further investigated. Our analysis of bulk samples provides a good starting point to define the functional and environmental associations of the MES flavors, but single-cell approaches and experimental work would ultimately be needed to confirm these findings.

While previous studies defined MES as one of a few primary cellular states in glioblastoma, here we have highlighted the diversity that exists within MES states. In principle, any state could be further broken down into more subtle states, raising the question of what should be the resolution by which we describe cellular states. We would argue that it is useful to consider multiple different resolutions and that any attempt to conclude on a specific number of states will, on one hand, be incomplete, but on the other hand, will be too detailed for certain purposes. Thus, a primary classification of cells may only focus on separating malignant cells from other tumor-infiltrating cell types, a second classification of the malignant cells may focus on the four states that we previously defined [4], and a third layer of classification may focus on the flavors of each of those four states. The relevance of this third layer depends primarily on whether the different flavors are not only distinct in their gene expression profile but also in their genetic, environmental, or clinical associations. Indeed, we have shown here that the two extreme flavors of MES states have distinct associations with genetic (NF1) and environmental (hypoxia and immune cell types) features, and may have distinct clinical implications in the context of immunotherapies, together highlighting the significance of their distinction for advancing our understanding of glioma.

## Conclusions

Our work integrates multiple GBM datasets to define a core MES program and highlight three main flavors of MES states with distinct functional, genetic, and environmental associations. These states primarily differ in their association with hypoxia vs. astrocytic programs, and with immune suppression vs. activation, respectively. This diversity of MES states can bridge between the distinct associations suggested by previous studies, and the signatures defined here can serve as the basis for further studies of MES states.

## Supplementary Information

**Additional file 1: Table S1.** MES signatures from published studies. **Table S2.** Cohorts details. **Table S3.** Functional MES signatures. **Table S4.** Top-scoring genes from PCA analysis.

**Additional file 2: Figure S1.** Comparing core MES scores between studies, related to Fig. 1. **Figure S2.** Investigating the three MES states, related to Fig. 2. **Figure S3.** Correlations of MES states and macrophage states, related to Fig. 3. **Figure S4.** Correlations of MES states and T cell states, related to Fig. 4.

## Data Availability

This work did not produce new data. The code for this work is available online at https://github.com/rchanoch/MES_GBM_GENOME_MED [35].

## Abbreviations

GBM: Glioblastoma
EMT: Epithelial-to-mesenchymal transition
scRNA-seq: Single-cell RNA-seq
MES: Mesenchymal
TCGA: The Cancer Genome Atlas
IVY-GAP: Ivy Glioblastoma Atlas Project
MES-Hyp: Hypoxia-related mesenchymal state
MES-Ast: Astrocyte-related mesenchymal state
