# Correction: Elucidating the diversity of malignant mesenchymal states in glioblastoma by integrative analysis

**DOI:** 10.1186/s13073-022-01122-x

**Published:** 2022-10-12

**Authors:** Rony Chanoch-Myers, Adi Wider, Mario L. Suva, Itay Tirosh

**Affiliations:** 1 Department of Molecular Cell Biology, Weizmann Institute of Science, Rehovot, Israel; 2 Department of Pathology and Center for Cancer Research, Massachusetts General Hospital, Boston, MA USA; 3 Broad Institute of MIT and Harvard, Cambridge, MA USA

**Correction: Genome Med 14, 106 (2022)**

**https://doi.org/10.1186/s13073-022-01109-8**

The original publication of this article [1] contained an incorrect version of Fig. 1 which could not be amended before publication.

**Fig. 1:**
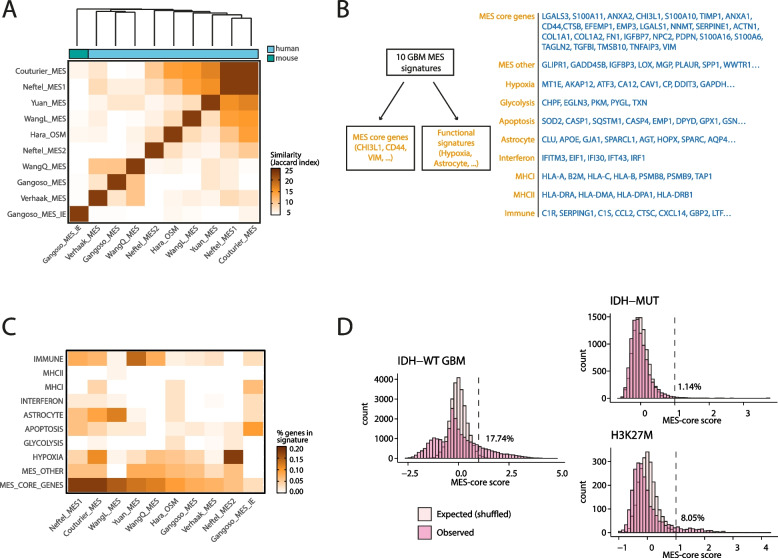
Correct version of figure 1. The full caption is available in the original article

The incorrect and correct version of Fig. 1 are shown in this correction article as figure 1 & 2 respectively, the original article has been updated.

**Fig. 2:**
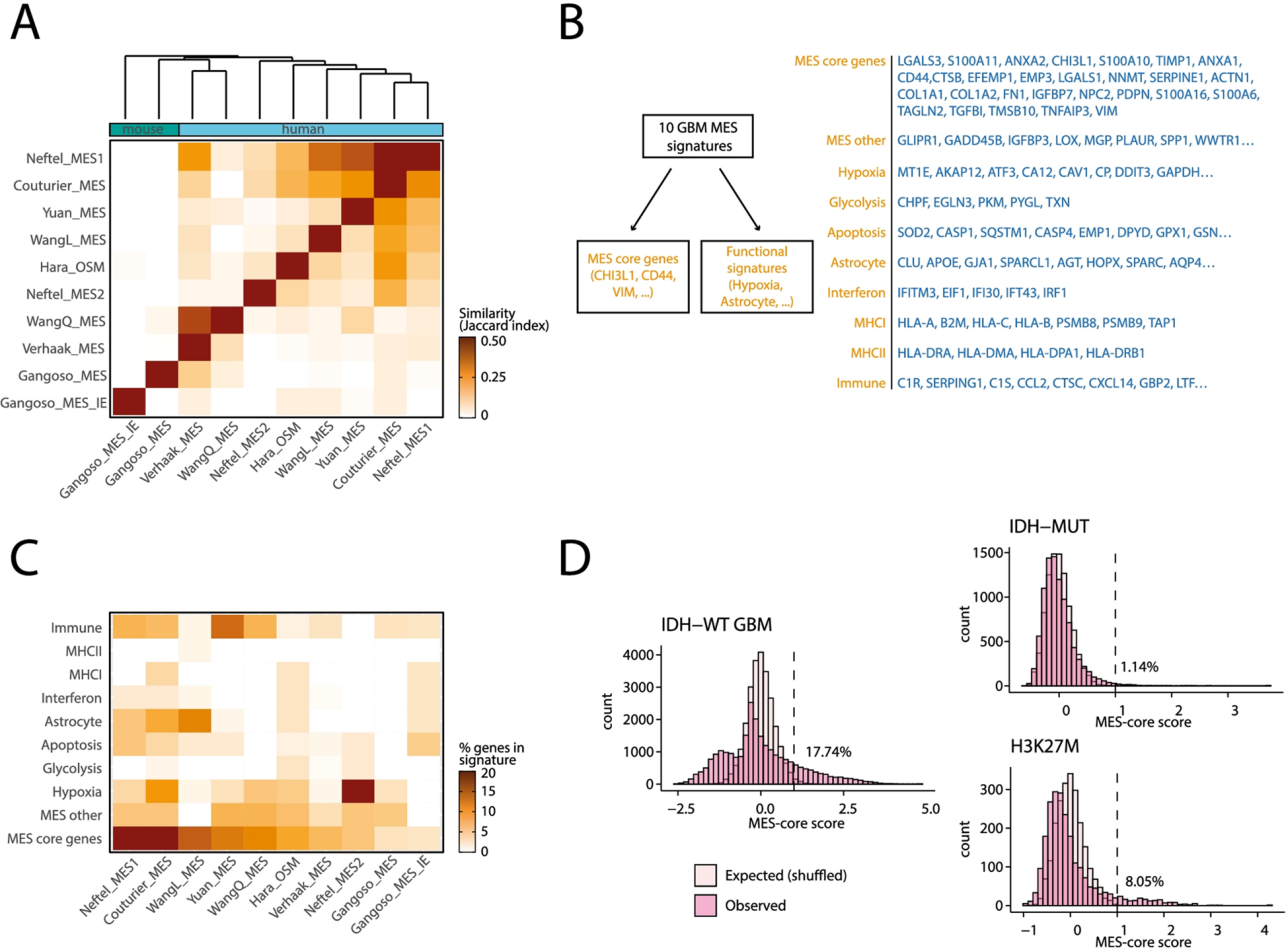
Incorrect version of figure 1
