# Supplementary material for: Elucidating the diversity of malignant mesenchymal states in glioblastoma by integrative analysis
Source: Genome Med. 2022 Sep 19;14:106. doi: 10.1186/s13073-022-01109-8 (PMC9484143; doi:10.1186/s13073-022-01109-8)
Supplement: Supplementary file 2 — Additional file 2: Figure S1. Comparing core MES scores between studies, related to Fig. 1. Figure S2. Investigating the three MES states, related to Fig. 2. Figure S3. Correlations of MES states and macrophage states, related to Fig. 3. Figure S4. Correlations of MES states and T cell states, related to Fig. 4. [file 13073_2022_1109_MOESM2_ESM.pdf]

Elucidating the diversity of malignant mesenchymal states in glioblastoma by integrative analysis

Additional file 2: Figures S1-S4

Figure Legends

Figure S1: Comparing core MES scores between studies, related to Figure 1. (A) Distributions of the MES-core scores of cells from the four IDH-WT GBM studies (pink). The light color represents the MES-core scores of the shuffled distributions. The threshold of MES cells (red dashed line) was calculated as the 99% quantile of the shuffled distribution for each study separately. (**B**) Distributions of average expression of the core MES program in cells from IDH-WT GBM. Malignant cells are colored in red and non-malignant cells are colored in pink, and core MES scores of the shuffled dataset colored in grey. (**C**) Heatmaps show the expression of MES-core program genes (y-axis) across all of the cells (x-axis) from the different glioma cohorts, ordered by their MES-core score. Cells before the vertical line passed the threshold as MES cells. (**D**) Kaplan-Meier curve of all high grade gliomas (top) or IDH-WT gliomas (bottom) from TCGA dataset, colored by Phillips et al subtype.

Figure S2: Investigating the three MES states, related to Figure 2. (A) Heatmap of all the GBM MES cells (x-axis), centered by their scores for the MES functional program (y-axis). The first row shows the average MES-core program score for each cell. Cells are ordered by the difference of the Hypoxia and Astrocyte programs, and vertical lines separate the cells into MES-Hyp, MES-intermediate, and MES-Ast. **(B)** Dot plots shows the PC1 and PC2 scores of MES cells from all of the samples from the each of the 4 GBM studies, colored by the number of genes detected per cell. **(C)** Dot plots show each cell's PC2 or PC3 score, and the difference of the Hypoxia and Astrocyte programs. **(D)** Heatmaps similar to S1A) that show the MES cells from individual tumors with higher average MES-Hyp scores (top), higher MES-Ast scores (bottom), or neither (middle). **(E)** Bottom: Heatmap shows the average score of each cell for the core MES, MES-Hyp, and MES-Ast programs in the different samples found in the Ivy-GAP dataset. Top: Annotation bar colored by the different locations each sample was extracted from. **(F)** Dot plot shows the expression of NF1 (X-axis) against the MES-core score (Y-axis) of RNA-seq GBM samples from TCGA.

Figure S3: Correlations of MES states and macrophage states, related to Figure 3. Dot plots show the **(A)** macrophage abundance score, **(B)** the difference of the macrophage and microglia score or (**C**) the difference of M1 and M1 activation score (X-axis) against the MES-core score (Y-axis) of RNA-seq GBM samples from TCGA.

Figure S4: Correlations of MES states and T cell states, related to Figure 4. Dot plots show the **(A)** T cell abundance score or **(B)** the difference of the cytotoxicity and exhaustion score (X-axis) against the MES-core score (Y-axis) of RNA-seq GBM samples from TCGA.

Figure S1

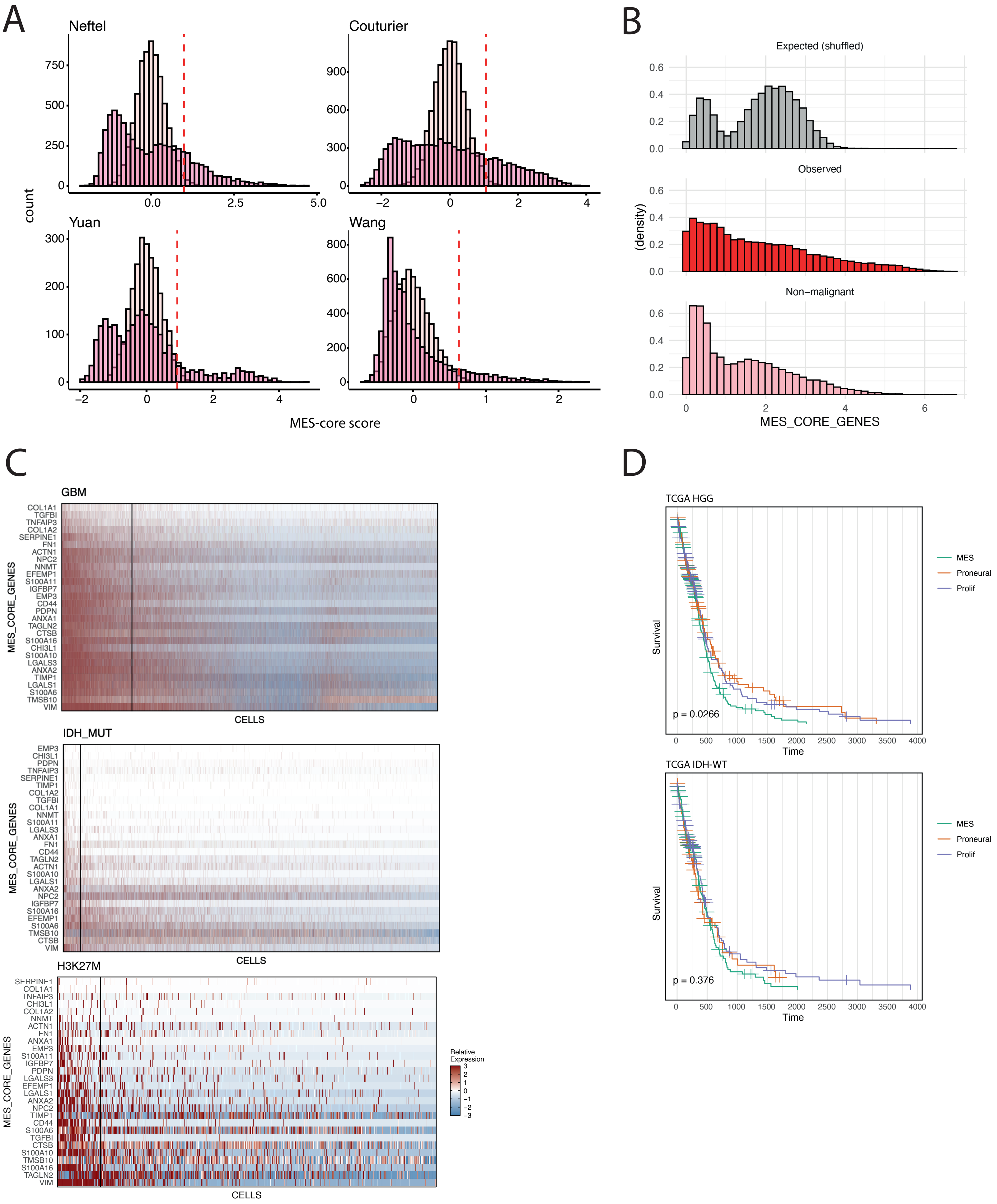
A
Neftel
Couturier
Yuan
Wang
count
MES-core score
B
Expected (shuffled)
Observed
Non−malignant
(density)
MES_CORE_GENES
C
GBM
MES_CORE_GENES
COL1A1
TGFBI
TNFAIP3
COL1A2
SERPINE1
FN1
ACTN1
NPC2
NNMT
EFEMP1
S100A11
IGFBP7
EMP3
CD44
PDPN
ANXA1
TAGLN2
CTSB
S100A16
CHI3L1
S100A10
LGALS3
ANXA2
TIMP1
LGALS1
S100A6
TMSB10
VIM
CELLS
IDH_MUT
EMP3
CHI3L1
PDPN
TNFAIP3
SERPINE1
TIMP1
COL1A2
TGFBI
COL1A1
NNMT
S100A11
LGALS3
ANXA1
FN1
CD44
TAGLN2
ACTN1
S100A10
LGALS1
ANXA2
NPC2
IGFBP7
S100A16
EFEMP1
S100A6
TMSB10
CTSB
VIM
CELLS
H3K27M
SERPINE1
COL1A1
TNFAIP3
CHI3L1
COL1A2
NNMT
ACTN1
FN1
ANXA1
EMP3
S100A11
IGFBP7
PDPN
LGALS3
EFEMP1
LGALS1
ANXA2
NPC2
TIMP1
CD44
S100A6
TGFBI
CTSB
S100A10
TMSB10
S100A16
TAGLN2
VIM
CELLS
Relative Expression
D
TCGA HGG
Survival
p = 0.0266
Time
MES
Proneural
Prolif
TCGA IDH-WT
Survival
p = 0.376
Time
MES
Proneural
Prolif

Figure S2

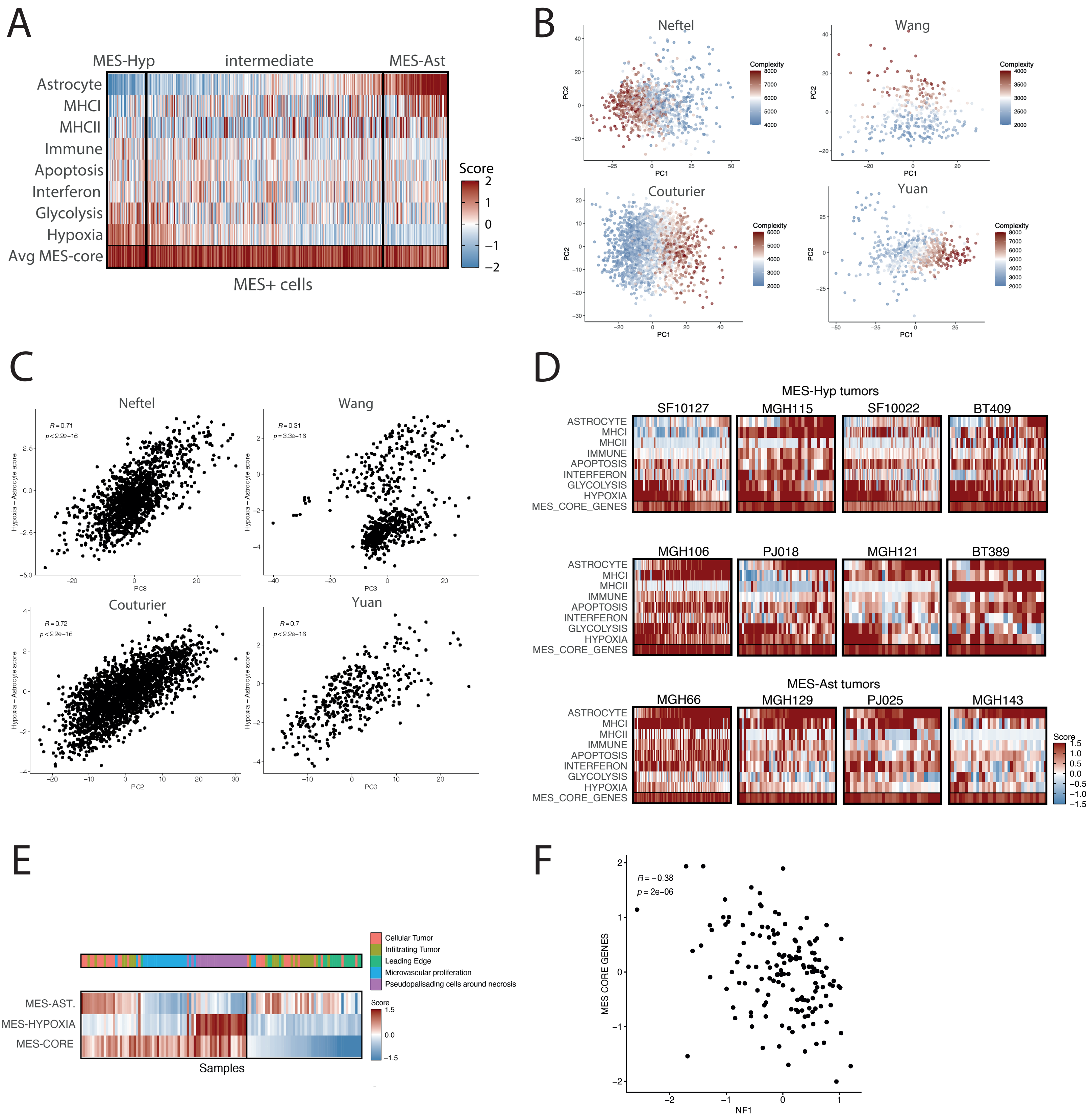
A
MES-Hyp
intermediate
MES-Ast
Astrocyte
MHCI
MHCII
Immune
Apoptosis
Interferon
Glycolysis
Hypoxia
Avg MES-core
Score
MES+ cells
B
Neftel
Wang
Couturier
Yuan
Complexity
PC1
PC2
C
R = 0.71
p < 2.2e−16
R = 0.31
p = 3.3e−16
R = 0.72
p < 2.2e−16
R = 0.7
p < 2.2e−16
Hypoxia − Astrocyte score
PC3
PC2
D
MES-Hyp tumors
SF10127
MGH115
SF10022
BT409
MGH106
PJ018
MGH121
BT389
MES-Ast tumors
MGH66
MGH129
PJ025
MGH143
ASTROCYTE
MHCI
MHCII
IMMUNE
APOPTOSIS
INTERFERON
GLYCOLYSIS
HYPOXIA
MES_CORE_GENES
Score
E
Cellular Tumor
Infiltrating Tumor
Leading Edge
Microvascular proliferation
Pseudopalisading cells around necrosis
MES-AST.
MES-HYPOXIA
MES-CORE
Score
Samples
F
R = −0.38
p = 2e−06
MES CORE GENES
NF1

Figure S3

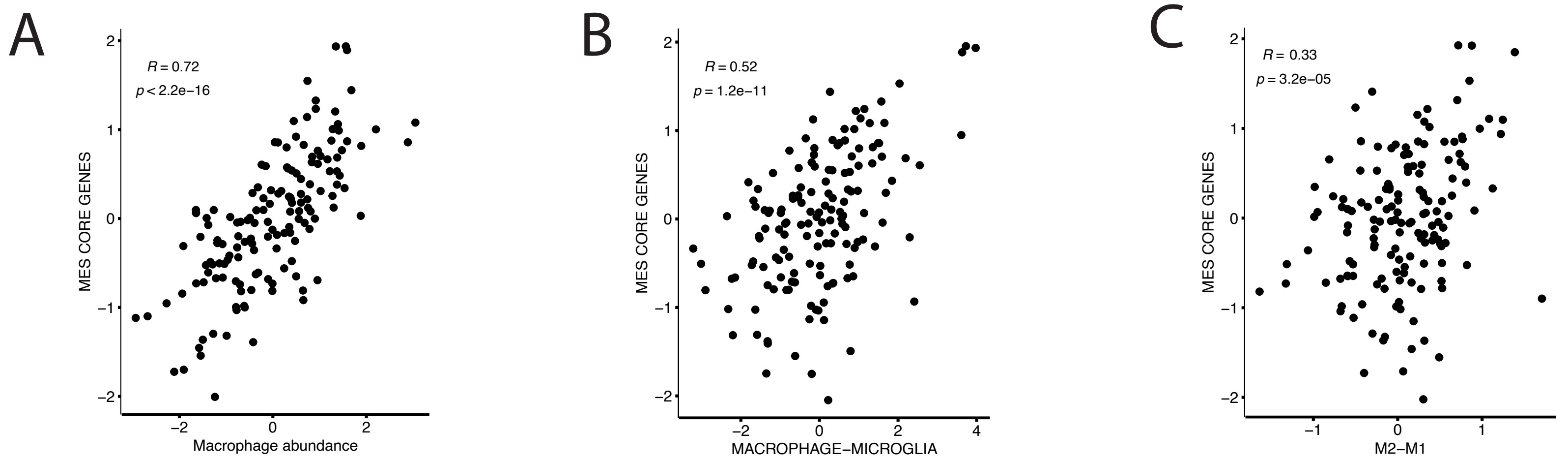
A
B
C
R = 0.72
p < 2.2e−16
R = 0.52
p = 1.2e−11
R = 0.33
p = 3.2e−05
MES CORE GENES
Macrophage abundance
MACROPHAGE−MICROGLIA
M2−M1

Figure S4

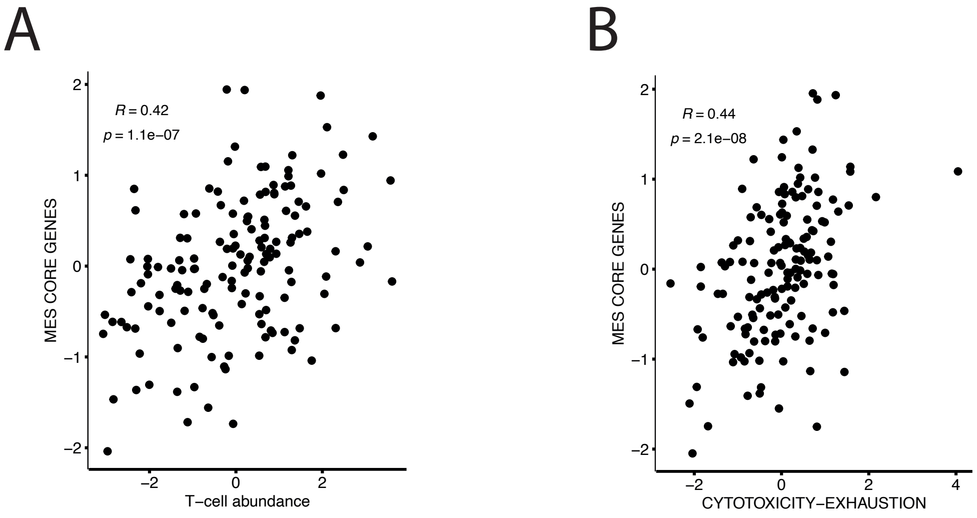
A
R = 0.42
p = 1.1e−07
MES CORE GENES
T−cell abundance
B
R = 0.44
p = 2.1e−08
MES CORE GENES
CYTOTOXICITY−EXHAUSTION
